# Discussion on Poliomyelitis

**Published:** 1916-12

**Authors:** Edward Clark

**Affiliations:** Buffalo, N. Y.; Sanitary Supervisor in the New York State Department of Health


### Discussion on Poliomyelitis.

Dr. EDWARD CLARK, Buffalo, N. Y.

Sanitary Supervisor in the New York State Department of Health.

In the early part of August, 1916, I was directed by Dr. Herman M. Biggs, State Commissioner of Health, to report at the Willard Parker Hospital in New York for the purpose of studying poliomyelitis. While at the Willard Parker Hospital I had an opportunity to see about 400 cases of poliomyelitis of various types; while there I also became familiar with their method of doing lumbar puncture work for purposes of confirmatory diagnosis. On the 15th of August I was directed to go to Middletown, Orange County, N. Y. to open a branch office of the State Department of Health from which the work of handling and controlling the poliomyelitis situation in Rockland, Orange, Sullivan and Ulster Counties was directed. In this work I was associated with Dr. Charles W. Berry and Dr. John A. Smith, Sanitary Supervisors in the State Department of Health. There were assigned to the Middletown office also three nurses who were engaged in doing field work. It may not be generally known, but the four counties above mentioned are the summer boarding house district for a large part of New York's great east side; it is estimated that five or six hundred thousand people, including a large number of children, spend a part of the summer in the above named counties, particularly in Ulster and Sullivan Counties. Sullivan County itself is largely the summer boarding district for the Jewish people from lower east New York.

In these counties up to the time of my leaving there had been reported approximately 480 cases of poliomyelitis. During the time of my connection with the office we had reported to us 264 cases, of these 152 were males and 112 females; 50.3 per cent of the cases occurred in children under 5 years of age; 32.3 per cent occurred in children between the ages of 5 and 10; 10.6 per cent occurred between the ages of 10 and 15; 3.1 per cent occurred between the ages of 15 and 20; 2.2 per cent occurred between the ages of 20 and 25; .74 per cent occurred between the ages of 29 and 30. Among adults over 30 years of age 3 cases occurred, all males, one 37 years of age, one 47 years of age, and one 51 years of age. The mortality rate in these four counties was about 20 per cent, the fatality seeming to increase with the ages of the patients. In the early part of the epidemic children mostly were affected, but as the epidemic progressed the cases among older persons seemed to increase.

The epidemic of poliomyelitis which has prevailed during the summer and fall of this year has been marked by two features which make it almost unique in the history of epidemics of this disease, these features are the widespread prevalence of the disease with the large number of cases reported, and the very high mortality rate. A study of the reports of different epidemics of poliomyelitis which have occurred during the past twenty years reveal the curious fact that the symptoms of the disease seem to vary largely in the different epidemics. In 1912 in the epidemic which prevailed in Western New York, I had an opportunity to observe quite a number of cases, and at that time gastro-intestinal disturbances marked by diarrhoea and catarrhal conditions of the nose and throat were quite common. In the recent epidemic these conditions were not frequently found. Of the 264 cases reported at Middletown office during my connection with it, 85 per cent had constipation, in many cases of a very obstinate type; about 90 per cent of the cases had marked rigidity of the neck, so that it was almost impossible to flex the head upon the sternum even to a slight extent without causing the patient severe pain. Drowsiness was a marked character in about 65 per cent of the cases. Nausea occurred in about 80 per cent of the cases, and vomiting occurred in about the same number of cases. The vomiting was not of the projectile type which we frequently see in cases of meningitis, but it was a vomiting which seemed to be induced by the administration of food or some unpleasant tasting medicine. Many of the patients would complain of a good deal of pain in the back and limbs upon being handled, but if left alone they did not complain of very much pain. All of the cases commenced with initial fever running all the way from 100 to 105 deg. Fahrenheit. Delirium occurred in a very small number of cases which I observed, and as a rule mentality was intact. Some authors have attached considerable importance to a peculiar and profuse sweating of the patient which was a marked symptom in some of the former epidemics. I did not observe this symptom except in a very few cases. Very few of the patients complained of sore throat, although the fauces and tonsils in many cases were very red. A peculiarly coated tongue was observed in a large number of cases, different from any coated tongue I have ever observed, and for want of a better name I called it the P. P. ''paste polic'' tongue on account of its peculiar appearance, particularly if seen early; the tongue looked as though ordinary office paste had been spread on it very carefully, just as you would spread butter on a slice of bread; later on the color changed and the papilae of the tongue would show around the edges. The Kernig sign was present in very many cases, either in both or one leg, the patellar reflexes early in the disease were sometimes exaggerated, later on they would be absent either on one or both sides.

The Babinski sign and the Brodzinski sign could be elicited in a small proportion of the cases, and in those cases in which there was a good deal of meningeal involvement with evidence of intra-cranial pressure, a positive MacEwen sign was occasionally observed.

In my experience the severity of the onset affords no criterion as to the amount of paralysis which will occur in a given case, many of the cases which had very high initial fever, where the patients were exceedingly nervous and irritable, would turn out to be of the abortive type of the disease and would quickly recover with no resulting paralysis. Different observers and writers have made a large number of classifications of different types of poliomyelitis, but I have put the cases which I have observed into three different classes, first the abortive, in which no resultant paralysis occurred; second the spinal type, third the bulbo-meningeal type. This last type takes in all the cases in which there seems to be a good deal of meningeal involvement and affects the nerve centers high up. In this type we would get quite a number of cases of facial paralysis, paralysis of the muscles of the eyes, paralysis of the muscles of the throat, etc.

Spinal puncture was resorted to in a number of cases as a therapeutic measure, particularly in those cases which showed the effects of cerebral involvement with inter-cranial pressure. This method was also resorted to in a number of cases for diagnostic purposes. The spinal fluid in poliomyelitis, if unmixed with blood, is always clear. If the case was one of poliomyelitis, and if the puncture was made early in the disease, an increase in the number of monouclear lymphocytes was observed. This increase was variable, ranging from 15 or 20 per cubic milimeter up to hundreds, the globulin or Noeguchi reaction was always present, and if the case was poliomyelitis a positive Fehling reduction for sugar was always observed. It is important in making examinations of spinal fluid for the cell count to see that the fluid is obtained in a thoroughly sterile manner and received in a sterilized test tube, and the cell count should be made just as quickly as possible after the fluid is withdrawn from the spinal canal. In order to do this we used a portable microscope, so that we had an opportunity to examine the fluid within a very few minutes after it was obtained.

I have talked with very many physicians who seem to labor under the impression that every case of poliomyelitis which occurs must necessarily be followed by more or less paralysis; this is an erroneous impression, because there are a great many of the so-called abortive cases, or as some would call them, pre-paralytic, which are cases of true poliomyelitis and can be diagnosed as such by a careful study of the clinical symptoms together with an examination of the spinal fluid. In most of the cases that proved fatal death was caused by paralysis of the muscles of respiration.

As I have not the time at my disposal I will not go into the question of the treatment of poliomyelitis at this time.

---

**Degenerate Family History.** Anna Wendt Finlayson of Warren, Pa., Bull. No. 15 of Eugenics Record Office, describes the descendents of Wm. and Mary Dack who emigrated from Ireland in 1815 and settled in western Pa. They were cousins and were both ignorant, the latter quarrelsome and the former a sheep thief. 754 descendents and -in-laws were traced. Exclusive of those dying under 20, -in-laws and those who moved away and could not be traced, 153 were left to be accounted for. None were ideal citizens but 40 were harmless and not a burden on society. 72 were shiftless, drunken, illiterate, quarrelsome, etc., but not criminal nor insane. 41 were either criminal or insane or otherwise showed marked indications of degeneracy. Otherwise classified, there were 25 insane, 20 shiftless, 39 below average intelligence, 34 ugly, 30 alcoholic, 27 notoriously irregular sexually, 18 had a habit of leaving their spouses. The worst lines were from two of the nine children of the original pair. One of these, a woman, married her cousin who was a ''bad man.'' The other, a man, married a defective woman of defective stock. The direct traceable cost to the state of this family has been $28,354, a small part of the total cost. (Note: Without in any way disparaging studies of this nature, it should be remembered that they tend to give an exaggerated conception of defective families. We have, for instance, no real standards of average families. If we took at random, 100 pairs of progenitors at 1815, the probability is that we would find comparatively few really valuable citizens, quite a number of shiftless, quarrelsome, drunken and mildly dishonest persons, while almost any family would show instances of sexual irregularity and one or two insane or criminal. The number who were in the habit of leaving their spouses is not far from the average. It represents about 1:9. allowing that all were married. Divorces average 1:12 marriages, and temporary separations are much more frequent).

